# An Enhanced Steganography Network for Concealing and Protecting Secret Image Data

**DOI:** 10.3390/e24091203

**Published:** 2022-08-28

**Authors:** Feng Chen, Qinghua Xing, Bing Sun, Xuehu Yan, Jingwen Cheng

**Affiliations:** 1 College of Air Defense and Anti-Missile, Air Force Engineering University, Xi’an 710051, China; 2 China Satellite Maritime Tracking and Control Department, Jiangyin 214430, China; 3 College of Electronic Engineering, National University of Defense Technology, Hefei 230037, China

**Keywords:** data protection, steganography network, computation complexity, non-activated feature fusion, imperceptibility, enhanced module

## Abstract

The development of Internet technology has provided great convenience for data transmission and sharing, but it also brings serious security problems that are related to data protection. As is detailed in this paper, an enhanced steganography network was designed to protect secret image data that contains private or confidential information; this network consists of a concealing network and a revealing network in order to achieve image embedding and recovery separately. To reduce the system’s computation complexity, we constructed the network’s framework using a down–up structure in order to compress the intermediate feature maps. In order to mitigate the input’s information loss caused by a sequence of convolution blocks, the long skip concatenation method was designed to pass the raw information to the top layer, thus synthesizing high-quality hidden images with fine texture details. In addition, we propose a novel strategy called non-activated feature fusion (NAFF), which is designed to provide stronger supervision for synthetizing higher-quality hidden images and recovered images. In order to further boost the hidden image’s visual quality and enhance its imperceptibility, an attention mechanism-based enhanced module was designed to reconstruct and enhance the salient target, thus covering up and obscuring the embedded secret content. Furthermore, a hybrid loss function that is composed of pixel domain loss and structure domain loss was designed to boost the hidden image’s structural quality and visual security. Our experimental results demonstrate that, due to the elaborate design of the network structure and loss function, our proposed method achieves high levels of imperceptibility and security.

## 1. Introduction

With the fast development of intelligent technology, the combination of information technology and intelligent technology has entered a rapid development stage [1]. Various interconnected information devices produce a large amount of image data every day. Some image data may contain sensitive or even confidential information, such as ID card images, bank card images, electronic images that are related to personal privacy, and even some confidential military images or remote sensing images. In recent years, digital media technology has provided great convenience for image processing, but it also brings serious security problems to the transmission of secret image data. The exposure of these secret image data in the public network will easily arouse the attention of malicious attackers, putting the secret image data at a high risk of being intercepted or stolen by criminals. Therefore, how to protect the secret image data and ensure their security has become an urgent issue.

Currently, encryption [2,3,4] is the most widely used security technique in the field of digital communication, as it usually leverages sophisticated and well-designed encryption algorithms to convert the secret information into a specific form of cipher-text. However, the encryption technique only focuses on the security of the information content; it cannot conceal the existence of secret information, which will arouse the attention and suspicion of third-party attackers. Even if the attackers cannot decipher the secret information in a short time, they can intercept the transmission process and locate the data terminals in order to perform an attack, which poses a great threat to the information’s transmission. In the past few years, a new security technique called digital steganography [5,6] has attracted wide attention and interest in both the academic community and the industrial field. Different from the encryption technique that makes the secret information unreadable and incomprehensible, the steganography technique aims to make the secret information imperceptible through concealing the secret information into a common carrier. The digital carriers that are used for steganography include binary text, audio and image, etc.; among them, the image is most widely used because it has large capacity for concealing more secret information and rich texture for enhancing the imperceptibility. Steganography using an image as a carrier is usually called image steganography [7,8], and many image steganography schemes have been proposed to achieve information hiding. We can divide these schemes into two categories, namely traditional image steganography and deep learning-based image steganography.

In traditional image steganography, the most typical representative method is the least significant bit (LSB) method [9]. As we all know, the image’s visual appearance is determined by its pixels, and each pixel contains three values of R, G and B. These pixel values are represented by an 8-bit binary, the image’s visual appearance is mainly determined by the highest bits and the lowest bits have the least influence on the visual appearance. Therefore, the LSB method is implemented through embedding secret information in the lowest bits of the cover image, so as to maintain the cover image’s visual appearance, unchanged. The LSB method is widely used because it can hide various forms of secret data, including plain text and image data; however, the secret data that are embedded by the LSB method are easy to detect due to the fixed manipulation and statistical property. Subsequently, another method called highly undetectable steganography (HUGO) was proposed [10], wherein the concept of a distortion function was first proposed for steganography modeling and guidance. Then, Holub et al. [11] proposed a method called wavelet obtained weights (WOW), which can adaptively embed the secret information into different image regions according to their textural complexity. In the work [12], Holub et al. further discussed a universal distortion function independent of the embedded domain, and the distortion function is defined as the sum of the relative changes of the coefficients of the multidirectional filter, which makes the modifications concentrate on the regions with rich texture or high-frequency noise, thereby avoiding embedding secret data into the smooth regions. In order to broaden the method’s application, a steganography method that was specially designed for JPEG images was proposed [13], which transforms the image into frequency domain using the discrete cosine transform. The distortion function is defined by the frequency coefficients that are available to flexibly allocate the embedded data.

Although the abovementioned traditional methods have greatly promoted the development of image steganography, they are still confronted with the following problems.

(1) They are always designed for concealing a small amount of bit-level messages (<0.4 bpp), where the payload capacity is measured in bits-per-pixel (bpp), thus they cannot meet the requirement of concealing the image data.
(2) They are always designed relying on domain knowledge and hand-crafted features with low dimensions, which cannot depict the target’s high-order statistical characteristics accurately [14], thus greatly limiting the steganographic performance. In addition, artificial design relying on domain knowledge is not only expensive, but also laborious and time-consuming.

In light of the above limitations of traditional steganography methods, in recent years, some scholars have explored introducing deep learning into the steganographic technique, striving to achieve a breakthrough over traditional methods by relying on the powerful feature representation ability of deep learning. Specifically, Tang et al. [15] designed an automatic learning framework that is based on generative adversarial networks (GAN), which produces a distortion probability map by training the discriminator against the generator. In order to generate higher-quality cover images, Shi et al. [16] designed a steganography network called SSGAN by replacing the traditional GAN with a novel WGAN [17], and they then adopted the traditional embedding algorithms in order to embed the secret data into the generated cover image. Although various methods [15,16] have made progress in their steganographic performance, they did not completely get rid of their dependence on traditional embedding algorithms. To this end, Zhu et al. [18] proposed an end-to-end steganography framework called HiDDeN, which can be applied in the fields of both steganography and watermarking. To further boost the payload capacity, a new steganography method called SteganoGAN [19] was proposed, and experiments show that this method achieved state-of-the-art payloads of 4.4 bpp. However, all of the abovementioned deep learning-based methods are still designed for concealing a small amount of bit-level messages. In order to achieve the goal of concealing image data, a novel encoder–decoder network [20] was designed based on convolutional neural networks (CNN), which can conceal a whole secret image into another cover image. However, this method generates hidden images with visible distortion due to the rough model design. In order to further improve the steganographic performance of this method, Duan et al. [21] proposed a deep reversible network and obtained a perceptually pleasing performance. However, the residual information between the hidden image and the original cover image, which largely determines the steganography’s security, was not demonstrated in their research. Subsequently, Baluja [22] displayed the residual map that was obtained by capturing the pixel-wise differences between the hidden image and the cover image and pointed out the research direction of improving steganography’s security; however, the hidden image’s quality has not been essentially improved. In order to further improve the hidden image’s quality and boost the steganographic performance from the source, Chen et al. [23] attempted to deepen and widen the network using various advanced feature fusion strategies and, as a result, a remarkable advancement was achieved.

Although the abovementioned deep learning-based methods [20,21,22,23] have successfully achieved the goal of concealing image data, the security issues, especially the visual security, have not been well solved. In addition, the recent studies usually design complex models in order to obtain performance improvement, but they ignore the model’s computational complexity in practical application.

Motivated by the above issues, this paper proposes a novel enhanced steganography network that is designed to conceal and protect secret image data. Compared with existing methods, our method not only eliminates the dependence on hand-crafted features, but also achieves high imperceptibility and security with low computational complexity.

Overall, the main contributions of this research are four-fold, as follows:

- We designed a novel steganography network for protecting secret image data, our simple but effective designs greatly improve the steganographic performance, almost without increasing the computation complexity, thus our method achieves high security with lower computational complexity when compared with existing methods.
- We propose the use of long skip concatenation in order to preserve more raw information, which greatly improves the hidden image’s quality. In addition, we propose a novel strategy, namely non-activated feature fusion (NAFF), in order to provide stronger supervision for synthetizing higher-quality hidden images and recovered images.
- We introduced the attention mechanism into image steganography and designed an enhanced module in order to reconstruct and enhance the hidden image’s salient target, which can effectively cover up and obscure the embedded secret content in order to enhance the visual security.
- We designed a hybrid steganography loss function that is composed of pixel domain loss and structure domain loss in order to comprehensively boost the hidden image’s quality, which greatly weakens the embedded secret content and enhances the visual security.

The rest of this article is structured as follows. Section 2 elaborates on our proposed method, including the specific network framework and the designed hybrid loss function. In Section 3, we present the experimental setup and demonstrate the performance of our proposed method. Our conclusions are presented in Section 4.

## 2. Methods

In this section, we first display the overall architecture of the proposed method and then give a detailed description about the specific framework components and our design considerations. After that, we introduce the designed hybrid steganography loss function.

### 2.1. Overall Architecture

**Figure 1:**
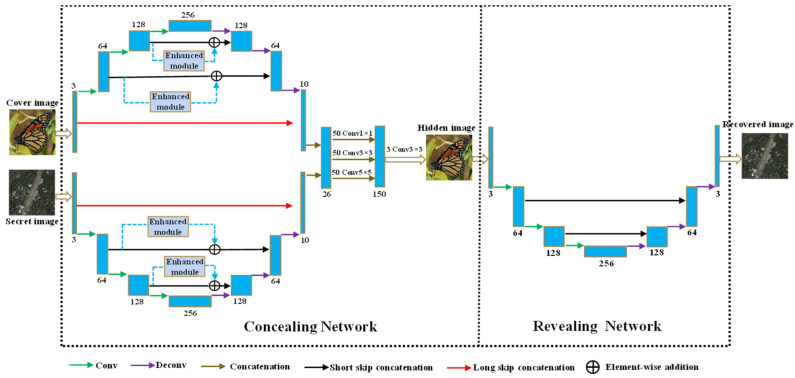
Overall architecture of the proposed method.

In previous image steganography methods, the researchers usually strive to improve the steganographic performance by relying on complex model design, which will greatly increase the method’s memory consumption. Considering the limited memory and computation resources in practical devices, such as smart phones, it is of great significance to design a low-complexity steganography model. For this purpose, we constructed the network with a down–up structure in order to reduce the resolution of the intermediate feature maps, thereby reducing the model’s complexity and memory overhead. The overall architecture of our proposed method is shown in Figure 1.

As is shown in Figure 1, the proposed network contains two sub-networks that are called the concealing network and the revealing network. First, the sender embeds the secret image into a common cover image using the concealing network and synthesizes an image called the hidden image (embedded with secret content). The hidden image is transmitted on the public Internet without arousing the suspicion of malicious attackers. Then, the receiver receives the hidden image and extracts the secret information from the hidden image using the revealing network in order to obtain a recovered image. In the concealing process, the cover image and the secret image are fed into two branches of the concealing network, separately. Through a series of down-sampling operations (that are achieved by continuous convolution layers), the input images are gradually compressed into small-scale feature maps. The down-sampling operation has the following advantages: (1) It can effectively capture high-level semantic information and long-range correlations from the pixel-wise information of the cover image and secret image. (2) It can reduce the resolution of the intermediate feature maps, thus reducing the model’s complexity and memory consumption. Subsequently, the resolution of the feature map is gradually expanded to the original image’s resolution through a series of up-sampling operations (that are achieved by implementing continuous deconvolution layers). Then, high-level abstract features extracted from the cover branch and the secret branch are merged in the top layer using the concatenation operation. Through multi-scale convolution operations of Conv 1 × 1, Conv 3 × 3 and Conv 5 × 5, rich features of different scales are further fully extracted and highly fused. Finally, the channel number of the feature map is reduced to 3 using the Conv 3 × 3 operation to obtain a hidden image that is visually similar to the cover image. Likewise, for the revealing network, its input is a hidden image embedded with secret information. Through a series of down-sampling operations, the resolution of the hidden image is gradually reduced and the valuable semantic information for reconstructing the secret image is extracted and preserved. Then, fine details of the secret image are gradually reconstructed by a series of up-sampling operations. The symmetrical convolution and deconvolution layers are connected by the short skip concatenation to enhance feature fusion of low-level features and high-level features, in addition, this operation can well fight the vanishing gradient problem. Numbers, such as 3, 26 and 50, represent the channel numbers of feature maps.

In this paper, each convolution and deconvolution layer is followed by batch normalization (BN) operation to speed up training except for the last layer of these two sub networks. In the down-sampling process, the LeakyReLU activation function with a slope of 0.2 is used and Relu activation function is used in the up-sampling process. The kernel size in the down-sampling and up-sampling process is set as 4 × 4 with the stride of 2. Unlike other studies that usually adopt the pooling operation for down-sampling, in our paper, the pooling operation is abandoned for down-sampling because it will lead to severe image details loss and increase the computation complexity.

### 2.2. Long Skip Concatenation

In our task, to ensure the imperceptibility, the synthetic hidden image is required to be similar to the cover image. In addition, to guarantee the recovery quality, the recovered image is expected to be similar to the secret image. However, after a series of convolution and deconvolution layers, the input images, such as the cover image and the secret image, will inevitably lose some information, which is extremely detrimental to the concealing and revealing performance. To alleviate this problem, we further propose the skip concatenation shown in Figure 1, which can directly pass the raw image information to the top layer and assist in synthesizing the edge and texture details, thereby improving the concealing and revealing performance.

The long skip concatenation is achieved using channel concatenation of feature maps, given an input image as $I_{i}$, the long skip concatenation can be described as

(1) $$I_{o}=F_{P}[I_{i}]*I_{i}$$

where $I_{o}$ represents the output of the concatenation operation, $F_{P}[]$ indicates the feature-extraction process implemented by a series of convolution–deconvolution layers, and ∗ represents the operation of channel concatenation. Suppose that the input image $I_{i}$ has $c_{1}$ channels and $F_{P}[I_{i}]$ has $c_{2}$ channels. After the concatenation operation, the output $I_{o}$ has $c_{1}+c_{2}$ channels. It can be observed that the long skip concatenation can directly pass the raw image information to the top layer and make the output $I_{o}$ contain the original input information $I_{i}$, thus effectively mitigating the information loss of the original input.

As shown in Figure 1, the cover image or the secret image only has 3 channels as R, G, and B, thus the long skip concatenation will not dramatically increase the channel numbers of the output feature maps. As a result, the computation complexity is only slightly increased.

### 2.3. NAFF Strategy

In previous computer vision tasks, such as object detection, target recognition and semantic segmentation, the activation function usually plays a significant role in the above tasks because the activated features represent higher-level features and attributes that are more conducive to distinguishing the high-level categories of the target. However, image-to-image steganography belongs to a mixed regime of high-level and low-level tasks, as it not only requires high-level semantic information to conduct the concealing and revealing process, but also requires low-level features to reconstruct the fine image details. However, the activation operation may modify the image’s detailed information and undermine the fidelity of some low-level features, such as the texture and edge information. For this reason, we propose a new strategy called NAFF to fuse the non-activated features, instead of the traditional activated features that are widely used in most computer vision studies. The proposed NAFF can preserve more low-level features of the cover and secret images and provide stronger supervision for synthesizing the texture and edge details, thereby improving the synthesis quality of the hidden image and the restoration quality of the secret image.

### 2.4. Enhanced Module

**Figure 2:**
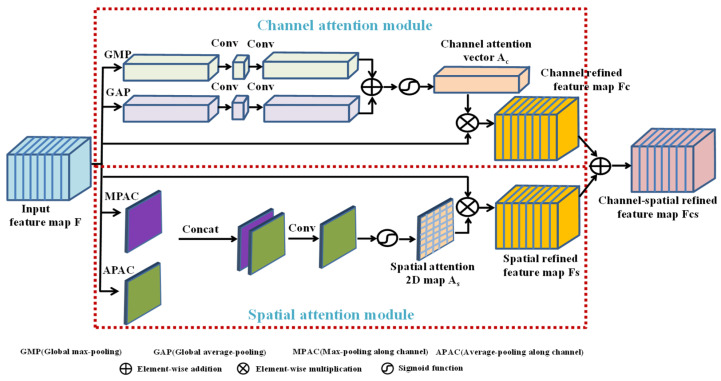
Architecture of the enhanced module.

The attention mechanism plays an important role in the perception of the human brain and human vision system, and it can conduct the perceptual system to focus on the salient region of the target object. In the past few years, the attention mechanism has been widely applied in various intelligent tasks, such as object detection [24], image captioning [25], etc., and achieved outstanding performance. Recently, some scholars attempted to introduce the attention mechanism into image steganography tasks to further improve the steganography performance. Specifically, Yu et al. [26] introduced the attention mechanism into GAN-based steganography without embedding and designed an attention module to focus on the spatial information and capture the correlation between pixels. Then, Tan et al. [27] proposed an end-to-end steganography network with an channel attention module to focus on the channel information and capture channel interdependencies. However, both methods [26,27] are still designed for concealing a small amount of bit-level messages. Inspired by the above works, we explore integrating the attention mechanism into our image steganography task for concealing image data, and we hope the attention mechanism will play two roles. The first role is to extract salient features from the cover image and synthesize the hidden image’s salient target to cover up and obscure the embedded secret content, thus enhancing the visual security and imperceptibility. The second role is to extract significant information from the secret image and preserve the important clues in the hidden image to restore the important secret content in the recovered image. Therefore, an enhanced module is designed to guide the network to adaptively focus on the salient region with significant features, which is shown in Figure 2.

Specifically, the enhanced module designed in this paper is composed of two sub-modules, namely a channel attention module and spatial attention module. These two sub-modules extract the salient features in parallel and focus on the salient features from two different perspectives of channel dimensions and spatial dimensions; thus, they are complementary to each other. Compared with the attention module [26] that only focuses on the spatial information and the attention module [27] that only focuses on the channel information, our designed enhanced module captures significant information in both the channel and spatial dimensions. Moreover, the designed enhanced module is super lightweight and greatly boosts the feature representation ability, almost without increasing the model’s complexity. Here, we give a detailed description of its modules.

(1) Channel attention module: To ensure the hidden image and the recovered image are visually similar to the original cover image and secret image, more attention should be paid to the salient regions, such as high-frequency texture, contour and edge that play crucial roles in the visual appearance. Therefore, global max-pooling (GMP) and global average-pooling (GAP), shown in Figure 2, are simultaneously applied to squeeze and aggregate the information from each channel of the input feature map. GMP can retain the most significant feature of all the channels, while GAP can be used as an auxiliary to gather other valuable information and retain the global information of all the channels. Assume that the input feature map $F\in R^{C\times H\times W}$ is fed into the channel attention module; after squeezing operations of GMP and GAP, two channel vectors with the size of $R^{C\times 1\times 1}$ can be obtained (Generally, the channel number represented by C is a large value, but the module’ complexity can be further reduced by using two continuous Conv 1 × 1 layers. The channel number of the first Conv 1 × 1 operation is C/r, where r is the reduction ratio of the channel number, and the channel number of the second Conv 1 × 1 operation is C. The Relu activation function is used between these two Conv 1 × 1 operations to further enhance the ability of extracting non-linear features. For simplicity, the above operations can be represented by a symbol called $C_{r}C$). Two channel vectors produced by $C_{r}C$ are added and then activated by the Sigmoid activation function to generate a channel attention vector as $A_{C}\in R^{C\times 1\times 1}$. Finally, we can obtain the channel refined feature map $F_{C}$ as follows:
  (2) $$\begin{array}{ll} F_{C} & =A_{C}⊗F\\ & =\delta (C_{r}C(\mathrm{GMP}(F))⊕C_{r}C(\mathrm{GAP}(F)))⊗F \end{array}$$
  where δ is the Sigmoid function; ⊗ and ⊕ indicate element-wise multiplication and element-wise addition, respectively.
(2) Spatial attention module: As shown in Figure 2, max-pooling along channel (MPAC) and average-pooling along channel (APAC) are simultaneously applied to extract the salient features and global information in the spatial dimension. Assume that the input feature map $F\in R^{C\times H\times W}$ is fed into the spatial attention module, and two 2D feature maps with the size of $R^{1\times H\times W}$ can be obtained after applying MPAC and APAC. Then, these two feature maps are concatenated into a hybrid feature map, which is then fed into a convolution layer with a Sigmoid activation function following to generate a spatial attention 2D map as $A_{S}\in R^{1\times H\times W}$. Finally, we can obtain the spatial refined feature map $F_{S}$ as follows:
  (3) $$\begin{array}{ll} F_{S} & =A_{S}⊗F\\ & =\delta (Conv(\mathrm{MPAC}(F)*\mathrm{APAC}(F)))⊗F \end{array}$$
  where ∗ represents the concatenation operation; Conv represents the convolution operation with the kernel size of 7 × 7, which means a large receptive field.

In conclusion, the channel-spatial refined feature map $F_{CS}$ is obtained as follows:

(4) $$\begin{array}{ll} F_{CS} & =F_{C}⊕F_{S}\\ & =\delta (C_{r}C(\mathrm{GMP}(F))⊕C_{r}C(\mathrm{GAP}(F)))⊗F⊕\\ & \;\delta (Conv(\mathrm{MPAC}(F)*\mathrm{APAC}(F)))⊗F \end{array}$$

In this paper, we adopt the residual connection [28] to integrate the enhanced module into the concealing network, as shown in Figure 1. The enhanced module can boost the ability of extracting salient features from the cover image and secret image, thus assisting in reconstructing the salient targets in the hidden image and the recovered image. The residual connection can preserve more raw information of the input feature map F. Combination of the raw information and the significant information extracted by the enhanced module can make the network adaptively select valuable information for improving the concealing and revealing performance. Finally, the residual enhanced feature map $F_{RCS}$ can be computed as follows:

(5) $$F_{RCS}=F_{CS}⊕F$$

### 2.5. Hybrid Loss Function

Currently, mean squared error (MSE) has become a dominant loss in various computer vision tasks. As stated in [29], relying on powerful feature representation ability, CNN has been widely applied to deal with various tasks, such as classification and regression tasks. In fact, different tasks with CNN do not necessarily rely on different network structures, and the main difference is reflected by the choice of the loss functions. The classification task generally adopts cross-entropy loss or Hinge loss as the loss function, while the regression task usually adopts MSE or root MSE as the loss function. Most of image steganography schemes also adopt MSE as the training loss for image synthesis. Therefore, in this paper, we adopt MSE as pixel domain loss (*PDL*), which is calculated across all of pixel values. Given two images x and y, the *PDL* is calculated as

(6) $$PDL(x,y)=\frac{1}{HW}\sum_{i=1}^{H}\sum_{j=1}^{W}{\left(x_{i,j}-y_{i,j}\right)}^{2}$$

where H and W represent the image height and width.

Although MSE can generate images with smooth consistency, it has been proven to generate perceptually unsatisfying images lacking of high-frequency details, which is particularly disadvantageous to the visual quality and imperceptibility. In previous studies, the structural similarity (SSIM) [30] is just used as a metric to evaluate image quality. In this paper, we attempt to introduce SSIM into steganography loss as structural domain loss (*SDL*), making the network focus on the structural quality, thus improving the visual quality and imperceptibility. We investigate the loss function for performance improvement and explore its relationship with the visual security. Given two images x and y, the *SDL* is calculated as

(7) $$SDL=1-\frac{\left(2u_{x}u_{y}+C_{1})(2\delta_{xy}+C_{2}\right)}{\left(u_{x}^{2}+u_{y}^{2}+C_{1})(\delta_{x}^{2}+\delta_{y}^{2}+C_{2}\right)}$$

where $u_{x}$ and $u_{y}$ represent the pixel means; $\delta_{x}^{2}$, $\delta_{y}^{2}$ and $\delta_{xy}$ denote the variances and the covariance; $C_{1}={\left(k_{1}L\right)}^{2}$, $C_{2}={\left(k_{2}L\right)}^{2}$; L denotes the max pixel value, set $k_{1}=0.01$ and $k_{2}=0.03$ by default [30].

Combine *PDL* and *SDL*, we design a hybrid loss function as

(8) $$\begin{array}{lll} Hybloss & = & Loss(H_{i,j},C_{i,j})+Loss(R_{i,j},S_{i,j})\\ & = & \left\{\alpha PDL(H_{i,j},C_{i,j})+\beta SDL(H_{i,j},C_{i,j})+\right\}+\\ & & \lambda \left\{\alpha PDL(R_{i,j},S_{i,j})+\beta SDL(R_{i,j},S_{i,j})\right\} \end{array}$$

where $H_{i,j}$ and $C_{i,j}$ represent the synthetic hidden image and the original cover image; $R_{i,j}$ and $S_{i,j}$ represent the recovered secret image and the original secret image. α, β are a group of parameters to balance the weight of *PDL* and *SDL*; λ is a hyper parameter to balance the weight of concealing loss and the revealing loss.

## 3. Experiments and Analysis

In this section, we first introduce the experimental platform and setup. Then, we perform extensive experiments to thoroughly verify the effectiveness of our design choices and demonstrate the superiority of our method.

### 3.1. Experimental Platform and Setup

Our experimental platform includes an Intel Xeon(R) Bronze 3104 CPU, and a NVIDIA GeForce RTX2080Ti GPU. Our method is carried out using PyTorch 1.3.0, which is one of the most popular deep learning frameworks.

To adaptively optimize the network more precisely, we use the ReduceLROnPlateau function to realize an automatic update of the learning rate, this function dynamically reduces the learning rate in the training process according to the measurement index, i.e., the hybrid loss function. Specifically, the parameters in the ReduceLROnPlateau function are set as factor=0.5, patience=5, which means that the learning rate will be automatically reduced when the loss function has not decreased for consecutive patience epochs, factor denotes the decline proportion of the learning rate. A computationally efficient optimizer called Adam optimizer with an initial learning rate of 0.001 is used in the experiment and the reduction ratio r is set as 16. α=1, β=0.2, and ξ=0.6 are set in this paper.

In this paper, VOC2007 [31] is used as the data set of cover images, NWPU-RESISC45 [32] is used as the data set of secret images. The training/testing ratio of both VOC2007 and NWPU-RESISC45 is set as 9:1. In the training process, the above two data sets are randomly shuffled to pair the cover images with secret images, thereby enhancing the diversity of matching styles.

### 3.2. Ablation Study

To verify the effectiveness and reliability of our design choices, we analyze their influence on the steganography performance by replacing or adding them for training. Here, we give the corresponding framework abbreviations, (1) BM: Baseline trained with the common MSE loss function (The baseline is composed of a stack of convolution and deconvolution layers with the short skip concatenation, which is shown in Figure 1); (2) BH: Baseline trained with the proposed hybrid loss function; (3) BHL: Baseline with hybrid loss function and long skip concatenation; (4) BHLN: Baseline with hybrid loss function, long skip concatenation and NAFF strategy; and (5) BHLNE: Baseline with hybrid loss function, long skip concatenation, NAFF strategy and the enhanced module. First, we use the same training set to train the abovementioned frameworks, then we verify these pre-trained frameworks using the same testing set. The average testing results are shown in Table 1.

Table 1 shows that our designed full model called BHLNE achieves the best concealing and revealing performance, both its generated hidden images and recovered secret images obtain the highest PNSR value and SSIM value, which indicates that both the hidden images and the recovered images produced by BHLNE have the highest quality. BM and BH show that, compared with the common MSE loss function, our designed hybrid loss function boosts the quality of both hidden images and recovered images, especially the SSIM value that largely determines the visual quality is greatly improved. BH and BHL indicate that, introducing the long skip concatenation effectively improves the concealing performance, but slightly reduces the revealing performance. This can be explained as our image steganography belonging to a double-object task and it is much more difficult than previous single-object tasks such as the super resolution task. Our image steganography task needs to achieve two goals; one is to make the hidden image appear similar to the original cover image, and the other is to make the recovered secret image appear similar to the original secret image. The concealing network and the revealing network interact with each other towards these two goals and obtain a trade-off between the concealing performance and revealing performance. Therefore, it is difficult to simultaneously achieve the two aforementioned goals perfectly. Nevertheless, it is still worthwhile to sacrifice a little revealing performance for a significant improvement of the concealing performance, because the concealing performance largely determines the steganography security. BHL and BHLN show that the proposed NAFF strategy that fuses the non-activated features further improves the quality of both hidden images and recovered secret images. BHLN and BHLNE demonstrate that integrating the enhanced module into the network can further boost the synthesis quality of the hidden image and the restoration quality of the secret image.

In addition, as shown in the last column of Table 1, our design choices effectively boost the concealing and revealing performance without significantly increasing the model’s computation complexity, except for the long skip concatenation that slightly increases the computation complexity.

**Figure 3:**
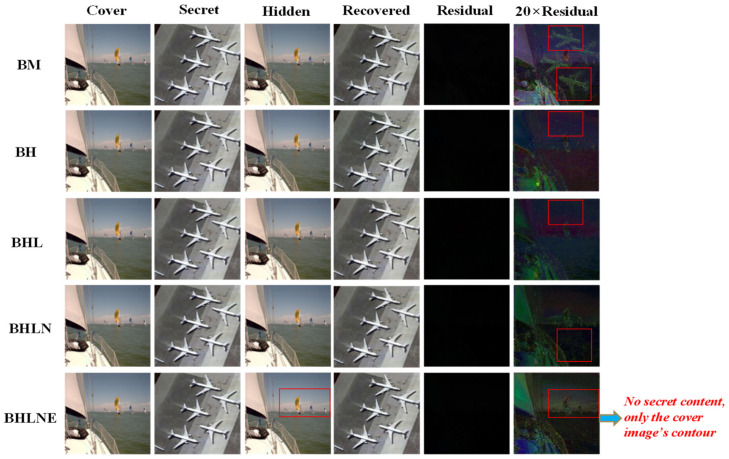
Visual ablation study of different frameworks.

To further intuitively show the influence of our design choices on the steganography performance, we demonstrate the visual ablation study in Figure 3. To guarantee a fair comparison, a pair of the same cover image and secret image is selected for visual verification.

In Figure 3, from left to right are the original cover image and secret image, the synthetic hidden image and the recovered secret image, the residual image (obtained by the pixel difference between the hidden image and the original cover image) and the 20× enhanced residual image. As shown in Figure 3, it is difficult to distinguish the performance difference caused by different elements just by observing the synthetic hidden image and the recovered secret image and even by the residual image, which indicates that our proposed baseline framework has achieved high concealing and revealing performance. However, after the residual image is magnified by 20 times, an interesting phenomenon occurs. BM shows that using traditional MSE as a steganography loss function will obviously expose the secret content in the 20× enhanced residual image, which obviously cannot meet the security requirement (Suppose that in practice, the malicious attackers on the Internet can easily obtain the original cover image from the public data set, then, they can decipher the secret content through the residual information between the hidden image and the cover image). BH indicates that the designed hybrid loss function plays a significant role in image steganography, it greatly improves the hidden image’s visual quality and weakens the secret content (highlighted by the red box) exposed in the 20× enhanced residual image, thus boosting the visual security. BHL and BHLN demonstrate that the long skip concatenation and the proposed NAFF strategy can further weaken the secret content exposed in the enhanced residual image and improve the visual security; however, there is still a slight secret trace exposed in the enhanced residual image, which is highlighted by the red box. As shown in the last row, i.e., BHLNE, the 20× enhanced residual image only displays the cover image’s salient target such as the sailboat highlighted by the red box, but does not display any secret content, which indicates that the hidden image generated by BHLNE has the highest imperceptibility and security. This can be explained that the proposed enhanced module can further boost the model’s ability to reconstruct and enhance the hidden image’s salient target, and the enhanced salient target can effectively cover up and obscure the embedded secret content, thus enhancing the imperceptibility.

### 3.3. Comparison Results

To comprehensively demonstrate the superiority of our proposed method, we compare it with the typical representative of traditional image steganography methods, namely LSB method, and the existing deep learning-based image-to-image steganography methods [20,21,22,23]. To guarantee a fair comparison, the abovementioned deep learning-based methods and our proposed method are all trained on the same training set and then tested on the same testing set. Table 2 displays the average testing results of the abovementioned methods.

It can be seen from Table 2 that, using the traditional LSB method to conceal image data generates hidden images and recovered images with poor quality, the average SSIM values of both the hidden images and recovered images are only about 0.90, and the average PSNR values are only about 30. Compared with the traditional steganography method, the recent deep leaning-based methods [20,21,22,23] greatly improve the quality of both the hidden image and the recovered secret image. However, as shown in the last row, our proposed method achieves the best concealing and revealing performance, both hidden images and recovered images achieve the highest SSIM value and PSNR value. More importantly, as shown in the last column, our proposed method achieves high steganography performance with low computation complexity. And its computation complexity is reduced about 60% when compared with the most recent method [23], which means that our method will have more probabilities to be applied in small devices, such as the smart phones.

**Figure 4:**
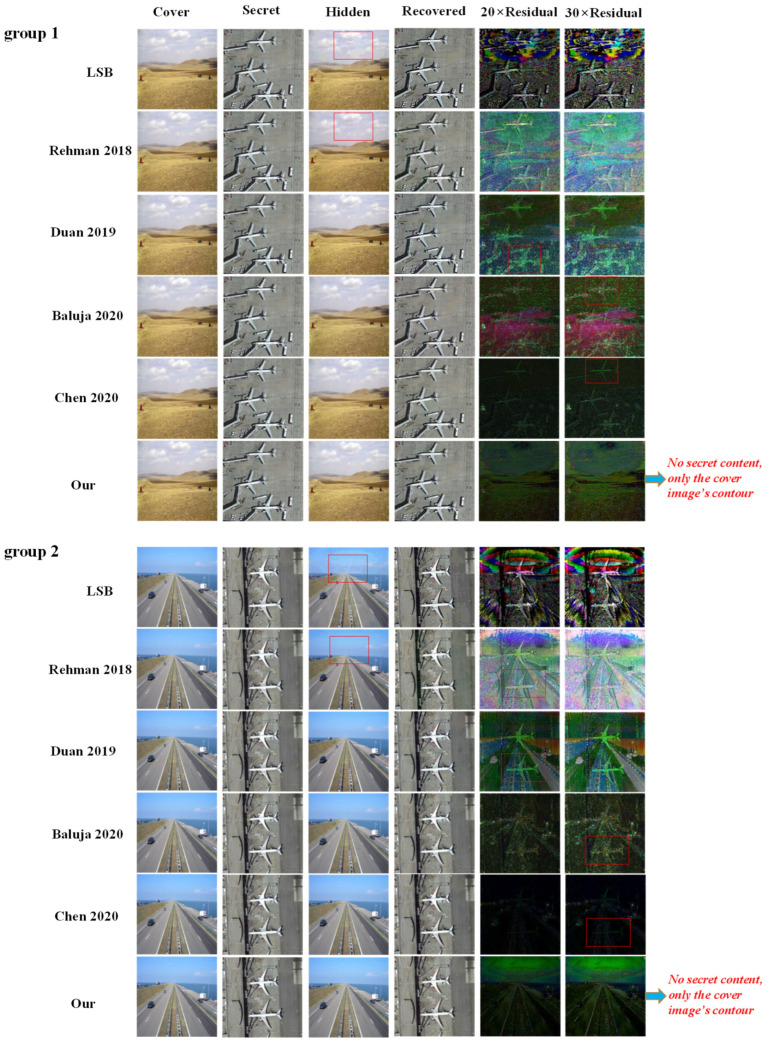
Visualization comparison between different methods.

To further intuitively demonstrate the visual performance difference between different methods, we display the visualization comparison in Figure 4. To ensure a fair comparison, we use the same cover images and secret images for visual verification. Due to the limitation of article space, here we only show two visualization examples.

From Figure 4, we can see that the traditional LSB method performs poorly when it is applied to conceal the large image data, and it produces obvious embedding traces in the hidden image, which is highlighted by the red box in the third column. Although the deep learning-based steganography method [20] gets rid of the dependence on traditional hand-crafted features, it still leaves slight modification traces in the hidden image due to the premature model design. Methods [21,22,23] further improve the concealing and revealing performance relying on advanced model design, as they can imperceptibly embed the secret image into the cover image and ensure the visual integrity of the hidden image, that is, there is no perceptible modification trace left in the hidden image. However, after the residual information between the hidden image and the cover image is magnified by 20 and 30 times, methods [21,22] largely expose the secret content in the enhanced residual images. Relying on complex model design, the most recent method [23] greatly improves the concealing performance and it only exposes a slight secret trace in the enhanced residual images. However, our proposed method obtains the best concealing performance with low computation complexity, it completely eliminates the secret content in the enhanced residual images and only displays the cover image’s contour to confuse the attackers, which means that even if the attackers would have access to the original cover image, no secret content could be found or deciphered through the residual information between the hidden image and the cover image. Therefore, the hidden image produced by our method has the highest security, which can well meet the security requirement in practical image transmission.

## 4. Conclusions

In this article, we propose an enhanced steganography network with low computation complexity to automatically protect the private or secret image data without relying on traditional embedding algorithms. We adopt a down–up structure to construct the overall architecture, thus reducing the model’s computation complexity. We propose the long skip concatenation to preserve more raw information and NAFF strategy to preserve more low-level features, which effectively improves the concealing performance. In addition, we further design an attention-based enhanced module to extract significant features from the original images, reconstructing and enhancing the salient target to cover up and obscure the embedded secret content, which effectively enhances the imperceptibility. For the steganography loss, we design a hybrid loss function to comprehensively boost the hidden image’s quality, thus significantly improving the visual security. More importantly, unlike other designs that rely on complex modules, our designs are simple and easy to implement, which effectively boosts the steganography performance, almost without increasing the computation complexity.

In practical application, the hidden image may suffer from noise and JPEG compressing attacks, and how to ensure the restoration integrity of the secret image under such extreme cases is our future research direction.

## Figures and Tables

**Table 1:** Average quantitative indicators of different frameworks.

Frameworks	PSNR $(H_{i,j}$ $C_{i,j})$	SSIM $(H_{i,j}$ $C_{i,j})$	PSNR $(R_{i,j}$ $S_{i,j})$	SSIM $(R_{i,j}$ $S_{i,j})$	Computation Complexity/GMac
BM	38.41	0.9841	35.57	0.9817	28.4
BH	38.57	0.9912	38.97	0.9914	28.4
BHL	40.46	0.9925	38.87	0.9912	29.1
BHLN	41.23	0.9937	38.92	0.9914	29.1
BHLNE	41.74	0.9941	39.23	0.9915	29.1

**Table 2:** Average quantitative indicators of different methods.

Methods	PSNR $(H_{i,j}$ $C_{i,j})$	SSIM $(H_{i,j}$ $C_{i,j})$	PSNR $(R_{i,j}$ $S_{i,j})$	SSIM $(R_{i,j}$ $S_{i,j})$	Computation Complexity/GMac
LSB	33.14	0.8980	27.75	0.9012	- -
Rehman [20]	33.53	0.9387	28.24	0.9332	3.1
Duan [21]	35.96	0.9514	35.34	0.9601	66.8
Baluja [22]	37.24	0.9608	35.78	0.9634	30.9
Chen [23]	41.51	0.9902	38.32	0.9842	87.6
Our	41.74	0.9941	39.23	0.9915	29.1

## Data Availability

Not applicable.
